# Prognostic and Immunomodulatory Roles of BNIP3 in Osteosarcoma Revealed by Integrated Single‐Cell and Bulk Transcriptomic Profiling

**DOI:** 10.1155/ijog/9272264

**Published:** 2026-03-08

**Authors:** Jiamiao Li, Wei Wang, Li Li, Kangjun Yu

**Affiliations:** ^1^ Department of Hand and Foot Surgery, Xianning Central Hospital, The First Affiliated Hospital of Hubei University of Science and Technology, Xianning, China

## Abstract

Osteosarcoma is a highly aggressive bone tumor with a complex tumor microenvironment (TME) that contributes to its progression and therapeutic resistance. In this study, we integrated single‐cell RNA sequencing (scRNA‐seq) and bulk RNA‐seq datasets to characterize the TME and identify key prognostic genes in osteosarcoma. Using scRNA‐seq data from 16 osteosarcoma samples, we defined eight major cell types within the TME and performed functional enrichment analyses. Through weighted gene co‐expression network analysis (WGCNA) focused on an inflammation‐related gene signature, we identified the yellow module as the most correlated with inflammation. By intersecting tumor‐upregulated genes with WGCNA‐derived genes, we identified BNIP3 as the only significant prognostic gene associated with poor survival in both the TARGET and GSE21257 cohorts. Functional annotation revealed that high BNIP3 expression is negatively correlated with immune‐related pathways and immune cell infiltration, including T cells, B cells, NK cells, and neutrophils. Additionally, BNIP3‐high patients exhibited a reduced sensitivity to several potential therapeutic agents. Our findings highlight BNIP3 as a hazardous gene in osteosarcoma, with important roles in immune evasion and prognosis, suggesting its potential as a therapeutic target.

## 1. Introduction

Osteosarcoma, the most prevalent primary malignant bone tumor in children and adolescents, presents a significant clinical challenge due to its aggressive nature, high metastatic potential, and unsatisfactory long‐term survival rates despite advances in multimodal therapies, including surgery and chemotherapy [1]. Its pronounced heterogeneity, both at the genetic and cellular levels, contributes to therapeutic resistance and disease relapse, underscoring the urgent need for a deeper molecular understanding to identify novel prognostic markers and therapeutic targets [2].

The tumor microenvironment (TME) is now recognized as a critical orchestrator of osteosarcoma progression, metastasis, and treatment response [3]. It is a complex ecosystem comprising tumor cells, immune cells (e.g., T cells, macrophages, and myeloid‐derived suppressor cells), stromal cells (including fibroblasts and endothelial cells), and noncellular components. Dynamic interactions within the TME can foster an immunosuppressive milieu, promote angiogenesis, and facilitate tumor cell invasion [4]. Among the hallmarks of cancer, inflammation plays a particularly pivotal role in shaping the TME, influencing immune cell recruitment, activation, and function, thereby affecting patient prognosis [5]. Immunotherapies, such as immune checkpoint inhibitors, have shown limited efficacy in osteosarcoma, which is often characterized as an immunologically “cold” tumor with a low immune cell infiltration and a suppressive TME. This highlights the critical need to identify key drivers of immune evasion. However, a precise, high‐resolution dissection of the inflammatory landscape and its key regulatory genes within the osteosarcoma TME remains incomplete.

The advent of single‐cell RNA sequencing (scRNA‐seq) technology has revolutionized our ability to deconvolute this complexity, enabling the characterization of cellular diversity, identification of rare cell populations, and inference of cell–cell communication at an unprecedented resolution [6]. Concurrently, bulk transcriptomic analyses of large patient cohorts provide robust statistical power for linking molecular features to clinical outcomes [7]. Integrating these complementary approaches offers a powerful strategy to bridge cellular phenotypes with patient‐level prognosis and to discover genes that are not only differentially expressed but also functionally central within disease‐relevant networks.

In this study, we leveraged integrated scRNA‐seq data from 16 osteosarcoma samples to comprehensively map the cellular composition and functional states of the TME. We combined this with bulk RNA‐seq data from the TARGET and GEO databases to perform a systems biology analysis. Specifically, we applied weighted gene co‐expression network analysis (WGCNA) to define gene modules co‐regulated with an inflammation‐related gene signature. By intersecting module genes with those upregulated in tumor cells, we aim to pinpoint key drivers situated at the nexus of tumor cell intrinsic properties and the inflammatory microenvironment. Our analysis identified BNIP3 (BCL2 interacting Protein 3), a gene involved in autophagy, apoptosis, and mitochondrial function, as a top candidate [8]. We subsequently validated its prognostic significance and conducted in‐depth explorations of its association with the immune landscape and drug sensitivity in osteosarcoma. This work establishes BNIP3 as a critical immunomodulatory prognostic factor, providing new insights into the pathogenesis of osteosarcoma and highlighting a potential target for therapeutic intervention.

## 2. Materials and Methods

### 2.1. Data Collection

The scRNA‐seq datasets, GSE152048 [9] and GSE162454 [10], of osteosarcoma were collected from the GEO database. The bulk RNA sequencing dataset of osteosarcoma was collected from the TARGET database and the GEO database (GSE21257) [11], respectively. The bulk RNA sequencing dataset of pan‐cancer data was collected from the TCGA database.

### 2.2. Data Analysis

The “inflammation”‐related gene list was obtained from the HALLMARK database [12]. The R package Seurat [13] was implemented to process the scRNA‐seq dataset. We systematically addressed batch effects using the Harmony algorithm. For quality control, cells were filtered to retain those with 200 < nFeature_RNA < 6000 and mitochondrial content of < 20%. The R package InferCNV was used to define tumor cells. Uniform manifold approximation and projection (UMAP) was used to present the distribution of cells. We used manual cell annotation based on canonical markers. The pathway enrichment analysis based on GOBP, KEGG, and WikiPathway terms of microenvironment cells was conducted using the R package SCP. The R package WGCNA was used to identify the “inflammation”‐related genes [14]. The connection between BNIP3 and stromal score, immune score, ESTIMATE score, and tumor purity using the ESTIMATE algorithm [15, 16]. The correlation between BNIP3 and immune cells was predicted using Porpimol′s study [17]. The drug prediction of BNIP3 was performed using the R package pRRophetic [18] based on the pharmacogenomic data from the GDSC database.

### 2.3. Statistical Analysis

All statistical analyses were performed using R and relevant bioinformatics packages. All *p* values were two‐sided, and statistical significance was set at *p* < 0.05 unless otherwise specified.

## 3. Results

### 3.1. Definition and Functional Annotation of Microenvironment Cells in Osteosarcoma at the scRNA‐seq Level

**Figure 2:**
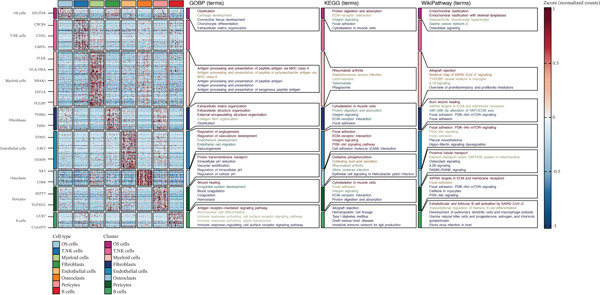
Functional annotation of microenvironment cells in osteosarcoma at the scRNA‐seq level. Heat map shows the pathway enrichment results of microenvironment cells based on GOBP, KEGG, and WikiPathway terms.

The integration of 16 osteosarcoma samples is shown in Figure 1a. The immune cells and nonimmune cells in the microenvironment of osteosarcoma were defined (Figure 1b). Subsequently, eight cell types, including tumor cells, T/NK cells, myeloid cells, fibroblasts, endothelial cells, osteoclasts, pericytes, and B cells, in the microenvironment of osteosarcoma were defined (Figure 1c). The pathway enrichment results based on GOBP, KEGG, and WikiPathway terms indicated that extracellular matrix organization, ECM–receptor interaction, and ossification were related to tumor cells (Figure 2). The enrichment of the “gastric cancer network” likely reflects the shared oncogenic signaling pathways common across cancers rather than tissue‐specific biology.

Figure 1Definition of microenvironment cells in osteosarcoma at the scRNA‐seq level. (a) UMAP shows the integration of 16 osteosarcoma samples. (b) UMAP shows the major cell categories in the microenvironment of osteosarcoma. (c) UMAP shows the minor cell categories in the microenvironment of osteosarcoma.

**Figure:**
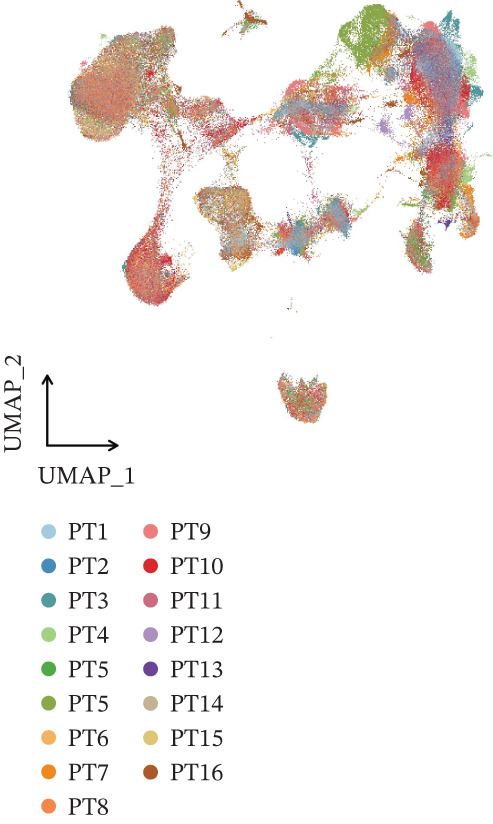
(a)

**Figure:**
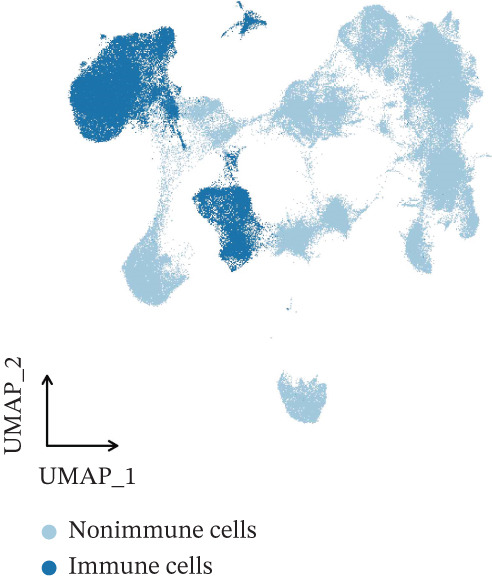
(b)

**Figure:**
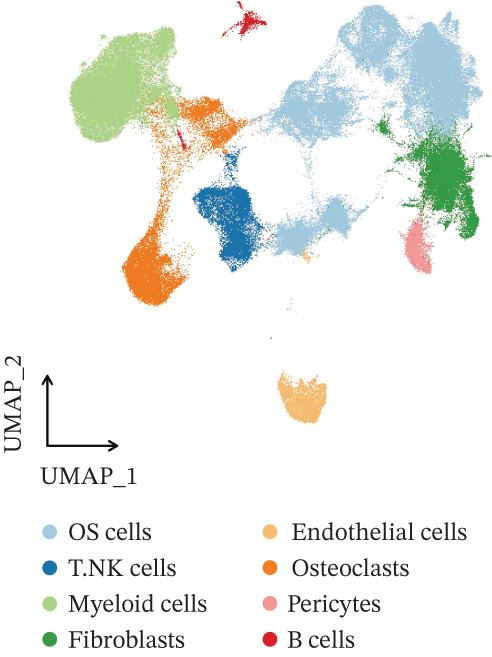
(c)

### 3.2. WGCNA on “Inflammation” Signature in Osteosarcoma

The optimal scale‐free topology model was determined at the soft threshold of four (Figure 3a). The dendrogram of the hierarchy of gene modules linked to the “inflammation” signature is shown in Figure 3b. The “inflammation” signature was found to correlate most with the yellow module (*R* = 0.73, *P* = 4*e* − 15) (Figure 3c). A significant association between gene significance and module membership in the yellow module is shown in Figure 3d.

Figure 3WGCNA on “inflammation” signature in osteosarcoma. (a) The association between scale‐free topology model fit, mean connectivity, and soft threshold. (b) Dendrogram shows the hierarchy of gene modules linked to the “inflammation” signature. (c) The correlation between “inflammation” signature and gene modules. (d) The association between gene significance and module membership in the yellow module.

**Figure:**
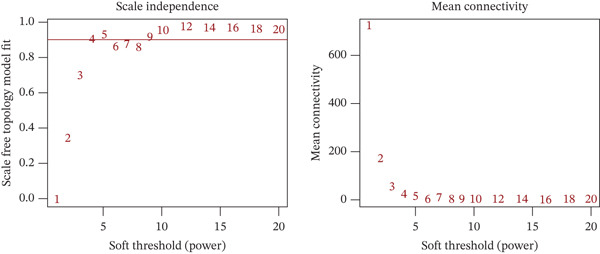
(a)

**Figure:**
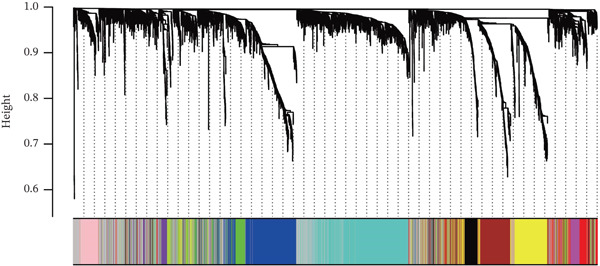
(b)

**Figure:**
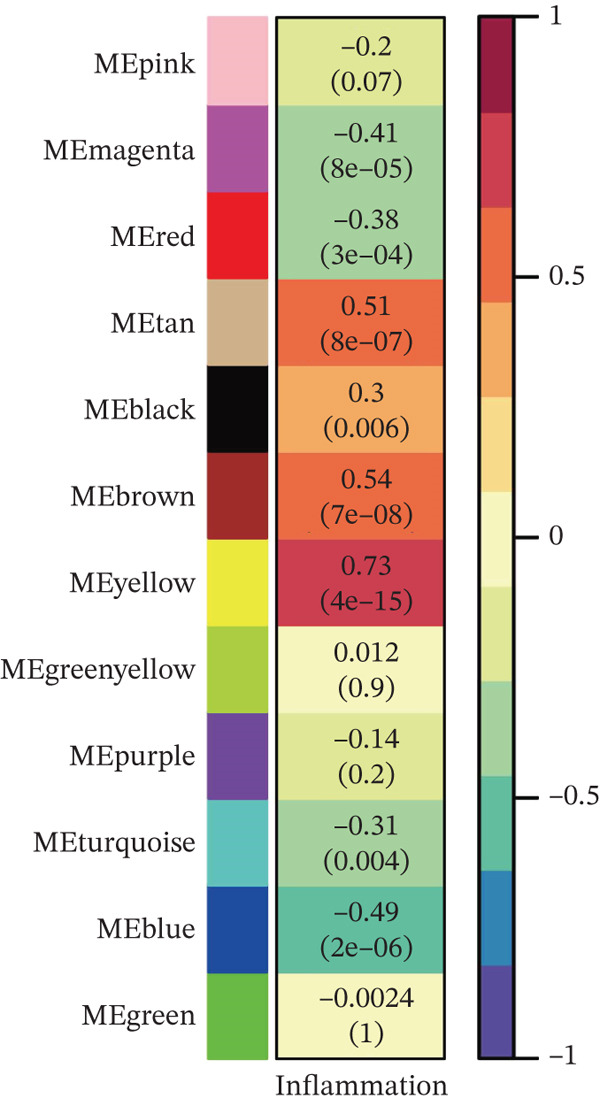
(c)

**Figure:**
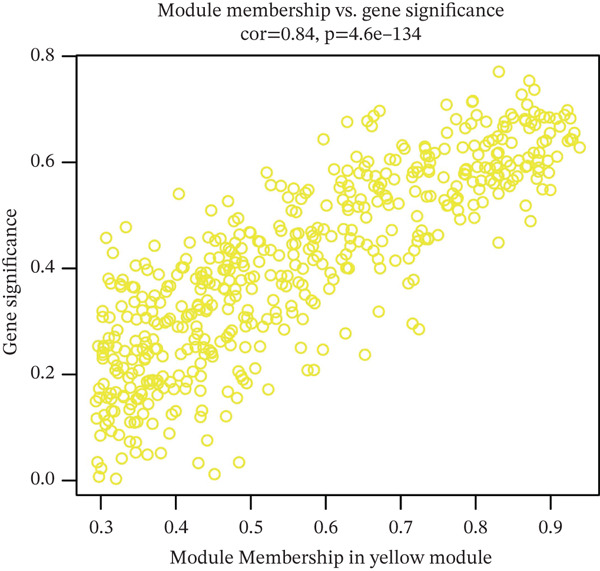
(d)

### 3.3. Identification of BNIP3 as a Hazardous Gene in Osteosarcoma

Tumor‐upregulated genes were defined by comparing the tumor cell cluster with all other nonmalignant cell clusters in the integrated scRNA‐seq data. Two hundred ninety‐eight upregulated genes in tumor cells compared with other microenvironment cells were identified. Four hundred ninety‐eight yellow module‐derived genes were extracted. Eight intersected genes between genes upregulated in tumor cells and WGCNA‐derived genes were finally identified (Figure 4a). Univariate Cox regression analysis confirmed BNIP3 to be the only prognostic intersected gene (*p* < 0.05) (Figure 4b). Accordingly, osteosarcoma patients with high BNIP3 expression were associated with worse survival outcomes in the TARGET cohort with a *p* < 0.0001 (Figure 4c) and the GSE21257 cohort with a *p* = 0.031 (Figure 4d).

Figure 4Identification of BNIP3 as a hazardous gene in osteosarcoma. (a) Venn plot shows the intersected genes between genes upregulated in tumor cells and WGCNA‐derived genes. (b) Univariate Cox regression analysis on eight intersected genes. (c) Survival curves of BNIP3‐based groups in the TARGET cohort. (d) Survival curves of BNIP3‐based groups in the GSE21257 cohort.

**Figure:**
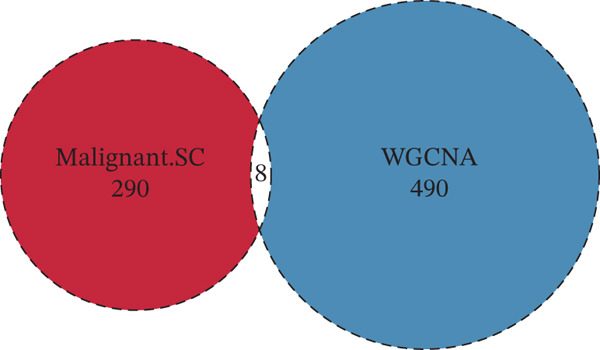
(a)

**Figure:**
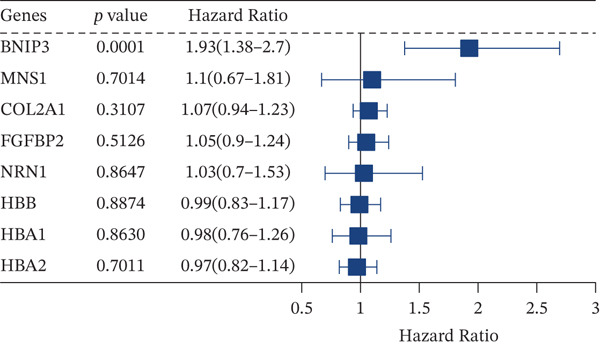
(b)

**Figure:**
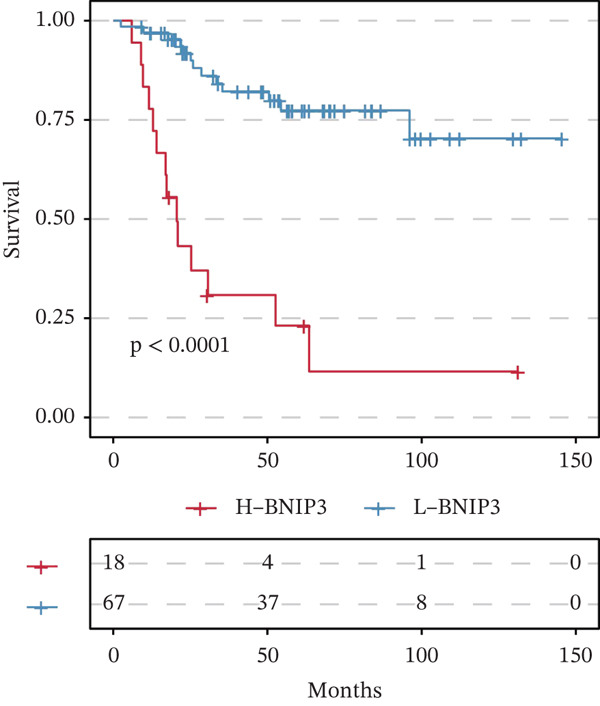
(c)

**Figure:**
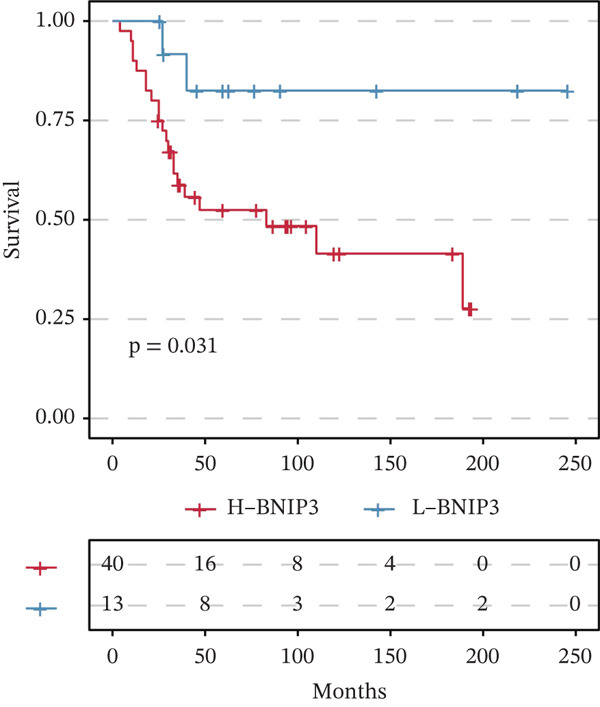
(d)

### 3.4. Drug Prediction of BNIP3 in Osteosarcoma

**Figure 5:**
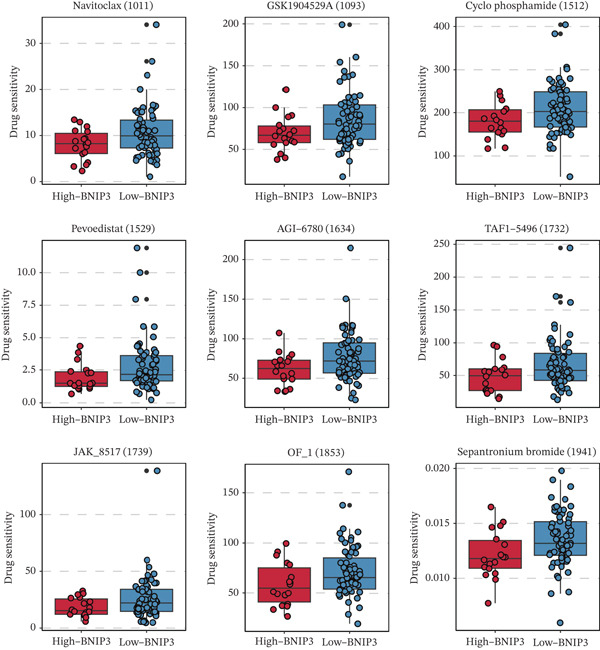
Drug prediction of BNIP3 in osteosarcoma. The drug sensitivity of nine drugs in BNIP3‐based groups in the TARGET cohort.

The drug sensitivity of nine drugs, including Navitoclax (1011), GSK1904529A (1093), cyclophosphamide (1512), Pevonedistat (1529), AGI‐6780 (1634), TAF1_5496 (1732), JAK_8517 (1739), OF‐1 (1853), and sepantronium bromide (1941), was significantly lower in osteosarcoma patients with high BNIP3 expression in the TARGET cohort (Figure 5). These results represent computational predictions that require experimental validation.

### 3.5. Functional Annotation of BNIP3 in Osteosarcoma

**Figure 6:**
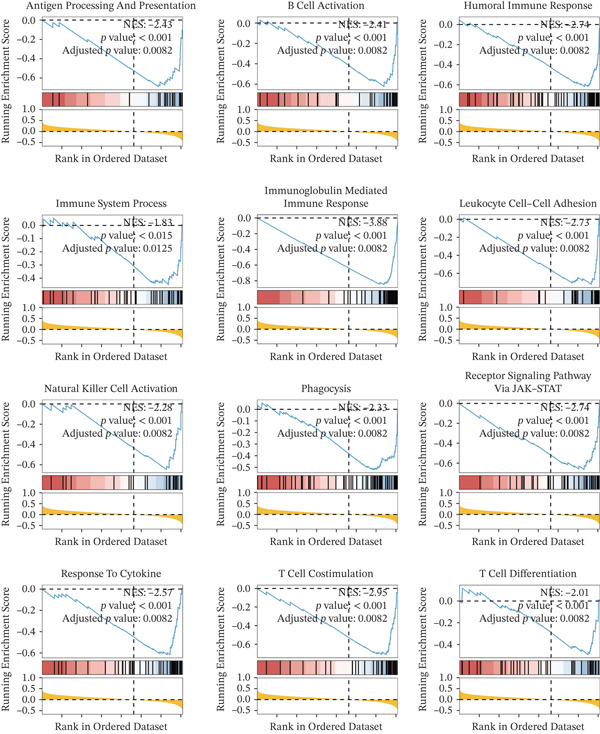
Functional annotation of BNIP3 in osteosarcoma. GSEA of 12 GO terms related to BNIP3 in the TARGET cohort.

Twelve GO‐based pathways, including T cell activity, B cell activity, NK cell activity, and immune response, were significantly negatively related to BNIP3 in the TARGET cohort (Figure 6).

### 3.6. Immune Characteristics of BNIP3 in Osteosarcoma

BNIP3 exhibited a significantly negative correlation with stromal score, immune score, ESTIMATE score, and tumor purity (Figure 7a). Additionally, immune cells, including T cells, B cells, NK cells, and neutrophils, were all negatively correlated with BNIP3 expression (Figure 7b).

Figure 7Immune characteristics of BNIP3 in osteosarcoma. (a) The correlation between BNIP3 and microenvironment scores. (b) Heat map shows the correlation between BNIP3 and immune cells.

**Figure:**
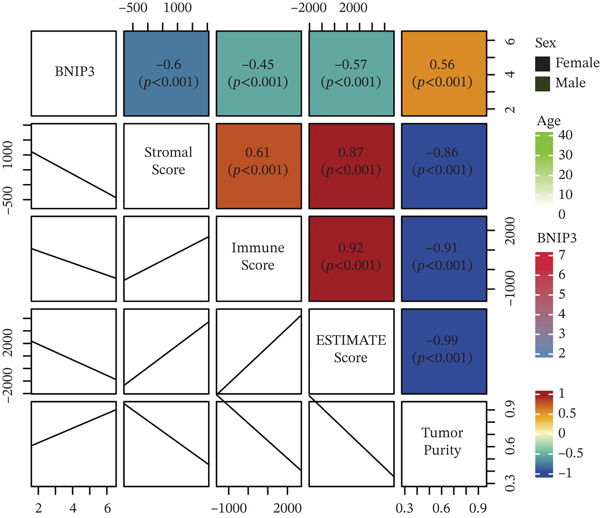
(a)

**Figure:**
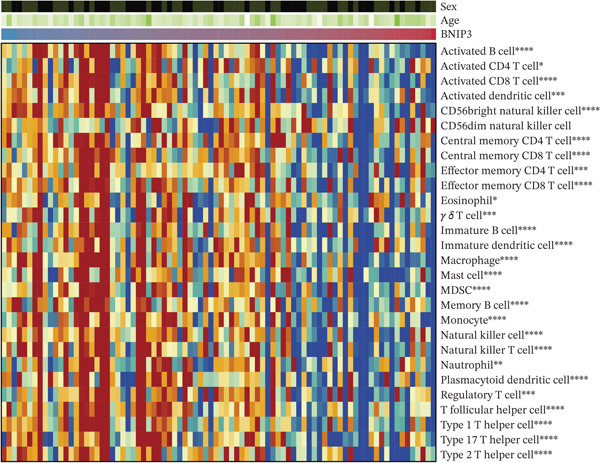
(b)

### 3.7. Pan‐Cancer Survival Analysis of BNIP3 Across 33 Tumor Types

**Figure 8:**
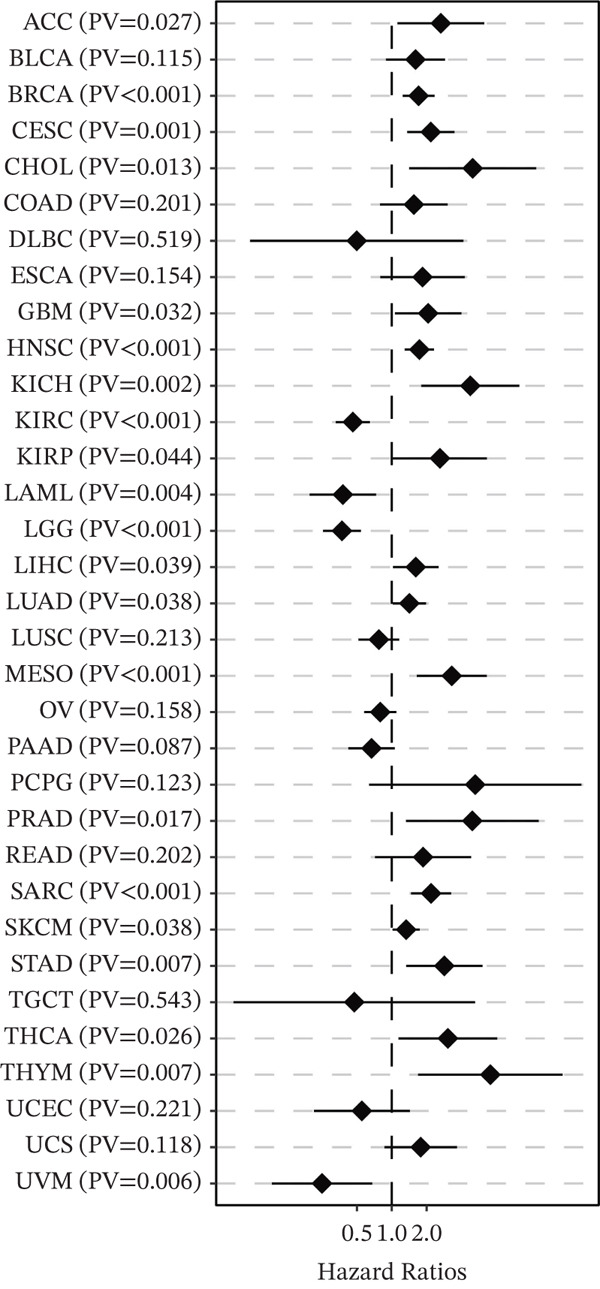
Pan‐cancer survival analysis of BNIP3 across 33 tumor types.

To investigate whether the prognostic value of BNIP3 observed in osteosarcoma is consistent across other malignancies, we extended our analysis to a pan‐cancer cohort using the TCGA database (Figure 8). The forest plot revealed that BNIP3 expression significantly correlates with overall survival in multiple cancer types. Consistent with our findings in the bone microenvironment (SARC), BNIP3 was identified as a significant risk factor in other major solid tumors, including breast invasive carcinoma (BRCA) and skin cutaneous melanoma (SKCM). This suggests that the adverse prognostic role of BNIP3 is not limited to osteosarcoma but may share underlying mechanisms with these aggressive epithelial and melanocytic tumors.

## 4. Discussion

Our study provides a comprehensive transcriptomic analysis of the osteosarcoma TME at single‐cell resolution, identifying BNIP3 as a novel prognostic and immunomodulatory gene. The integration of scRNA‐seq data allowed us to define eight major cell types within the TME, including tumor cells, immune subsets, and stromal components, highlighting the cellular complexity of osteosarcoma. Through WGCNA, we identified an inflammation‐related gene signature associated with the yellow module, which was enriched for genes involved in immune and stromal regulation. BNIP3 emerged as a key intersection gene between tumor‐upregulated genes and the inflammation‐associated module, and it was the only gene significantly associated with poor survival in two independent cohorts. This underscores its potential as a robust prognostic biomarker in osteosarcoma [19].

The exclusive significance of BNIP3 among the intersected candidates suggests it occupies a critical functional nexus‐linking tumor cell biology and inflammatory signaling. Functionally, BNIP3 expression was negatively correlated with multiple immune‐related pathways and immune cell infiltration, suggesting its role in creating an immunosuppressive TME. The negative association with stromal, immune, and ESTIMATE scores further supports the idea that BNIP3 may contribute to a “cold” tumor immune phenotype, which is often linked to resistance to immunotherapy [20]. We hypothesize that BNIP3‐mediated autophagy and mitophagy in osteosarcoma cells may facilitate immune evasion through several potential mechanisms: (1) by reducing mitochondrial reactive oxygen species (ROS) and associated immunogenic cell death; (2) by limiting the release of damage‐associated molecular patterns (DAMPs) and mitochondrial DNA that can stimulate antitumor immunity; and (3) by promoting metabolic adaptations that alter the immunomodulatory secretome of tumor cells, collectively impairing immune cell recruitment and function. These findings align with previous studies implicating BNIP3 in autophagy and apoptosis, but extend its role to immune modulation in osteosarcoma.

Moreover, drug sensitivity analysis revealed that high BNIP3 expression correlates with reduced sensitivity to several agents, including Navitoclax and cyclophosphamide, indicating potential mechanisms of chemoresistance. For Navitoclax, BNIP3′s interaction with BCL‐2 family proteins and its prosurvival mitophagic function may provide an escape pathway. For cyclophosphamide, which induces oxidative stress, BNIP3‐mediated clearance of damaged mitochondria could enhance tumor cell survival. This highlights the need for targeted therapeutic strategies that could overcome BNIP3‐mediated resistance [21, 22].

It is important to note that BNIP3′s role in cancer is context‐dependent, acting as either a prosurvival or prodeath factor in different malignancies. In osteosarcoma, our consistent findings across cohorts suggest its prosurvival, adaptive functions likely dominate, promoting tumor resilience and a suppressive TME. The observed association between high BNIP3 and poor prognosis in osteosarcoma appears to be consistent with its hazardous role in some other cancers like glioblastoma, though it can be favorable in contexts like colorectal cancer, underscoring the importance of the tissue‐specific microenvironment.

Limitations of this study include its retrospective nature and reliance on publicly available datasets. Furthermore, mRNA expression may not always correlate perfectly with protein activity, and the drug sensitivity predictions are based on pan‐cancer cell line data, which may not fully capture osteosarcoma‐specific biology. Functional validation through in vitro and in vivo experiments is required to confirm the mechanistic role of BNIP3 in osteosarcoma progression and immune evasion. Additionally, the clinical translation of BNIP3 as a biomarker or therapeutic target warrants further investigation in prospective cohorts. Future studies should also evaluate whether BNIP3 expression is an independent prognostic factor when accounting for clinical variables like metastasis.

## 5. Conclusion

In summary, our integrative multiomics analysis identifies BNIP3 as a critical prognostic biomarker and immunomodulatory gene in osteosarcoma. High BNIP3 expression is associated with poor survival, reduced immune infiltration, and altered drug sensitivity, highlighting its potential as a therapeutic target. Although no specific BNIP3 inhibitors are currently in clinical development, our findings highlight the need for future research to explore strategies targeting BNIP3 or its downstream pathways to overcome therapy resistance. These findings provide new insights into the inflammatory landscape of osteosarcoma and pave the way for future studies aimed at targeting BNIP3 to improve clinical outcomes.

## Author Contributions

Kangjun Yu and Li Li designed the study. Jiamiao Li and Wei Wang conducted analyses and drafted the paper.

## Funding

No funding was received for this manuscript.

## Disclosure

All authors critically reviewed and approved the final manuscript.

## Consent

The authors have nothing to report.

## Conflicts of Interest

The authors declare no conflicts of interest.

## Data Availability

All analytical data generated during this study are available from the corresponding authors upon reasonable request.

## References

[1] Meltzer P. S. and Helman L. J. , New Horizons in the Treatment of Osteosarcoma, New England Journal of Medicine. (2021) 385, no. 22, 2066–2076, 10.1056/NEJMra2103423, 34818481. 34818481

[2] Yu S. and Yao X. , Advances on Immunotherapy for Osteosarcoma, Molecular Cancer. (2024) 23, 10.1186/s12943-024-02105-9. PMC11382402 39245737

[3] Orrapin S. , Moonmuang S. , Udomruk S. , Yongpitakwattana P. , Pruksakorn D. , and Chaiyawat P. , Unlocking the Tumor-Immune Microenvironment in Osteosarcoma: Insights Into the Immune Landscape and Mechanisms, Frontiers in Immunology. (2024) 15, 10.3389/fimmu.2024.1394284. PMC11444963 39359731

[4] Wu A. , Yang Z. K. , Kong P. , Yu P. , Li Y. T. , Xu J. L. , Bian S. S. , and Teng J. W. , Exploring Osteosarcoma Based on the Tumor Microenvironment, Frontiers in Immunology. (2024) 15, 1423194, 10.3389/fimmu.2024.1423194. 39654890 PMC11625786

[5] Zhang R. , Zhou X. , and Shen C. , Inflammation-Driven Prognostic Model and Immune Landscape Profiling in Osteosarcoma, Discover Oncology. (2025) 16, 10.1007/s12672-025-03691-w. PMC12595213 41201557

[6] Papalexi E. and Satija R. , Single-Cell RNA Sequencing to Explore Immune Cell Heterogeneity, Nature Reviews. Immunology. (2018) 18, no. 1, 35–45, 10.1038/nri.2017.76, 2-s2.0-85039047649, 28787399. 28787399

[7] Hong M. , Tao S. , Zhang L. , Diao L. T. , Huang X. , Huang S. , Xie S. J. , Xiao Z. D. , and Zhang H. , RNA Sequencing: New Technologies and Applications in Cancer Research, Journal of Hematology & Oncology. (2020) 13, 10.1186/s13045-020-01005-x. PMC7716291 33276803

[8] Zhang J. and Ney P. A. , Role of BNIP3 and NIX in Cell Death, Autophagy, and Mitophagy, Cell Death and Differentiation. (2009) 16, no. 7, 939–946, 10.1038/cdd.2009.16, 2-s2.0-67549101188, 19229244. 19229244 PMC2768230

[9] Zhou Y. , Yang D. , Yang Q. , Lv X. , Huang W. , Zhou Z. , Wang Y. , Zhang Z. , Yuan T. , Ding X. , Tang L. , Zhang J. , Yin J. , Huang Y. , Yu W. , Wang Y. , Zhou C. , Su Y. , He A. , Sun Y. , Shen Z. , Qian B. , Meng W. , Fei J. , Yao Y. , Pan X. , Chen P. , and Hu H. , Single-Cell RNA Landscape of Intratumoral Heterogeneity and Immunosuppressive Microenvironment in Advanced Osteosarcoma, Nature Communications. (2020) 11, 10.1038/s41467-021-23119-7. PMC7730477 33303760

[10] Liu Y. , He M. , Tang H. , Xie T. , Lin Y. , Liu S. , Liang J. , Li F. , Luo K. , Yang M. , Teng H. , Luo X. , He J. , Liao S. , Huang Q. , Feng W. , Zhan X. , and Wei Q. , Single-Cell and Spatial Transcriptomics Reveal Metastasis Mechanism and Microenvironment Remodeling of Lymph Node in Osteosarcoma, BMC Medicine. (2024) 22, 10.1186/s12916-024-03319-w. PMC11100118 38755647

[11] Buddingh E. P. , Kuijjer M. L. , Duim R. A. , Burger H. , Agelopoulos K. , Myklebost O. , Serra M. , Mertens F. , Hogendoorn P. C. , Lankester A. C. , and Cleton-Jansen A. M. , Tumor-Infiltrating Macrophages Are Associated With Metastasis Suppression in High-Grade Osteosarcoma: A Rationale for Treatment With Macrophage Activating Agents, Clinical Cancer Research. (2011) 17, no. 8, 2110–2119, 10.1158/1078-0432.CCR-10-2047, 2-s2.0-79954573473, 21372215. 21372215

[12] Liberzon A. , Birger C. , Thorvaldsdottir H. , Ghandi M. , Mesirov J. P. , and Tamayo P. , The Molecular Signatures Database (MSigDB) Hallmark Gene Set Collection, Cell Systems. (2015) 1, no. 6, 417–425, 10.1016/j.cels.2015.12.004, 2-s2.0-84955328286, 26771021. 26771021 PMC4707969

[13] Satija R. , Farrell J. A. , Gennert D. , Schier A. F. , and Regev A. , Spatial Reconstruction of Single-Cell Gene Expression Data, Nature Biotechnology. (2015) 33, no. 5, 495–502, 10.1038/nbt.3192, 2-s2.0-84929151009, 25867923. PMC4430369 25867923

[14] Langfelder P. and Horvath S. , WGCNA: An R Package for Weighted Correlation Network Analysis, BMC Bioinformatics. (2008) 9, 10.1186/1471-2105-9-559, 2-s2.0-60549111634. PMC2631488 19114008

[15] Yoshihara K. , Shahmoradgoli M. , Martínez E. , Vegesna R. , Kim H. , Torres-Garcia W. , Treviño V. , Shen H. , Laird P. W. , Levine D. A. , Carter S. L. , Getz G. , Stemke-Hale K. , Mills G. B. , and Verhaak R. G. W. , Inferring Tumour Purity and Stromal and Immune Cell Admixture From Expression Data, Nature Communications. (2013) 4, no. 1, 10.1038/ncomms3612, 2-s2.0-84885673911. PMC3826632 24113773

[16] Zhang N. , Yang M. , Yang J. M. , Zhang C. Y. , and Guo A. Y. , A Predictive Network-Based Immune Checkpoint Blockade Immunotherapeutic Signature Optimizing Patient Selection and Treatment Strategies, Small Methods. (2024) 8, e2301685, 10.1002/smtd.202301685. 38546036

[17] Charoentong P. , Finotello F. , Angelova M. , Mayer C. , Efremova M. , Rieder D. , Hackl H. , and Trajanoski Z. , Pan-Cancer Immunogenomic Analyses Reveal Genotype-Immunophenotype Relationships and Predictors of Response to Checkpoint Blockade, Cell Reports. (2017) 18, no. 1, 248–262, 10.1016/j.celrep.2016.12.019, 2-s2.0-85009106590, 28052254. 28052254

[18] Geeleher P. , Cox N. , and Huang R. S. , pRRophetic: An R Package for Prediction of Clinical Chemotherapeutic Response From Tumor Gene Expression Levels, PLoS One. (2014) 9, e107468, 10.1371/journal.pone.0107468, 2-s2.0-84907212698. 25229481 PMC4167990

[19] Yu Q. , Fu W. , Fu Y. , Ye W. , Yan H. , Yu Z. , Li R. , Cai Y. , Chen Y. , Wang L. , Wei X. , Chen Y. , Zhang Y. , Ying H. , Tang F. , Dai F. , and Han W. , BNIP3 as a Potential Biomarker for the Identification of Prognosis and Diagnosis in Solid Tumours, Molecular Cancer. (2023) 22, 10.1186/s12943-023-01808-9. PMC10466744 37649051

[20] Romano E. , Rufo N. , Korf H. , Mathieu C. , Garg A. D. , and Agostinis P. , BNIP3 Modulates the Interface Between B16-F10 Melanoma Cells and Immune Cells, Oncotarget. (2018) 9, no. 25, 17631–17644, 10.18632/oncotarget.24815, 2-s2.0-85044839429, 29707136. 29707136 PMC5915144

[21] Cocetta V. , Vianello C. , Giacomello M. , and Montopoli M. , BNIP3-Dependent Mitophagy Role in Cisplatin Resistance, Autophagy Reports. (2022) 1, no. 1, 516–518, 10.1080/27694127.2022.2131210, 40396026. 40396026 PMC11864682

[22] Pan B. , Li Y. , Han H. , Zhang L. , Hu X. , Pan Y. , and Peng Z. , FoxG1/BNIP3 Axis Promotes Mitophagy and Blunts Cisplatin Resistance in Osteosarcoma, Cancer Science. (2024) 115, no. 8, 2565–2577, 10.1111/cas.16242, 38932521. 38932521 PMC11309937

